# The Community Primary Care Champions Fellowship: a mixed methods evaluation of an interprofessional fellowship for physician assistants and physicians

**DOI:** 10.1186/s12909-024-05559-z

**Published:** 2024-05-21

**Authors:** Shanna D. Stryker, Daniel Hargraves, Veronica Velasquez, Melissa Gottschlich, Patrick Cafferty, Darla Vale, Jeff Schlaudecker, Harini Pallerla, Megan Rich

**Affiliations:** 1 Department of Family and Community Medicine, University of Cincinnati College of Medicine, 231 Albert Sabin Way ML0582, Medical Sciences Building 4453C, Cincinnati, OH 45267 USA; 2 Department of Physician Assistant Studies, Mount St. Joseph University, Cincinnati, OH USA

**Keywords:** Interdisciplinary, Leadership, Faculty development, Graduate medical education, Continuing education, Team-based care, Family medicine, Internal medicine

## Abstract

**Background:**

Primary care in the US faces challenges with clinician recruitment, retention, and burnout, with further workforce shortages predicted in the next decade. Team-based care can be protective against clinician burnout, and opportunities for interprofessional education (IPE) on professional development and leadership could encourage primary care transformation. Despite an increasingly important role in the primary care workforce, IPE initiatives training physician assistants (PAs) alongside physicians are rare. We describe the design, curriculum, and outcomes from an interprofessional primary care transformation fellowship for community-based primary care physicians and PAs.

**Methods:**

The Community Primary Care Champions (CPCC) Fellowship was a one-year, part-time fellowship which trained nine PAs, fourteen physicians, and a behavioralist with at least two years of post-graduate clinical experience in six content pillars: quality improvement (QI), wellness and burnout, mental health, social determinants of health, medical education, and substance use disorders. The fellowship included a recurring schedule of monthly activities in self-study, lectures, mentoring, and community expert evening discussions. Evaluation of the fellowship included pre, post, and one-year follow-up self-assessments of knowledge, attitudes, and confidence in the six content areas, pre- and post- wellness surveys, lecture and discussion evaluations, and midpoint and exit focus groups.

**Results:**

Fellows showed significant improvement in 24 of 28 self-assessment items across all content areas post-fellowship, and in 16 of 18 items one-year post-fellowship. They demonstrated reductions in emotional exhaustion and depersonalization post**-**fellowship and increased confidence in working in interprofessional teams post-fellowship which persisted on one-year follow-up assessments. All fellows completed QI projects and four presented their work at national conferences. Focus group data showed that fellows experienced collaborative, meaningful professional development that was relevant to their clinical work. They appreciated the flexible format and inclusion of interprofessional community experts in evening discussions.

**Conclusions:**

The CPCC fellowship fostered an interprofessional community of practice that provided an effective IPE experience for physicians and PAs. The learning activities, and particularly the community expert discussions, allowed for a flexible, relevant experience, resulting in personal and professional growth along with increased confidence working within interprofessional teams.

## Background

A strong primary care system is essential to providing quality care at lower costs for patients in the United States [1]. However, US primary care currently faces many issues, including burnout and challenges with recruitment and retention of physicians [2–5]. There is a predicted shortage of over 68,000 primary care physicians in 2036, and a 37% shortage in nonmetro areas [3, 6]. Over the last several years, steps to improve primary care in the US have included modification of the triple aim into the quadruple aim to include provider wellness and promotion of the patient centered medical home (PCMH) model and the Center for Medicare and Medicaid Innovation’s Comprehensive Primary Care (CPC) program [7–9]. More recently the Quintuple Aim in health care has introduced health equity as its own pillar to improve both the care setting for health care professionals and the care and health of underrepresented and minoritized patient populations, further emphasizing the need for advocacy and leadership in primary care [10]. Additionally, given that close to 50% of clinicians in direct patient care experience burnout syndrome, and given an increasing aging population and complexity of patients, transformation of primary care is more critical than ever to meet the needs of patients and clinicians [3, 11, 12].

A key component for successful primary care transformation is interprofessional collaboration and team-based care [13, 14]. Team-based care has been shown to benefit clinical practice in various ways, including better patient outcomes, fewer medical errors, improved team efficiency and cohesiveness, and a positive impact on clinician well-being [15–21]. Furthermore, practice features that include team-based care addressing the social and behavioral health needs of patients, support quality improvement (QI) initiatives, and provide professional development and teaching opportunities, have been shown to be protective against primary care workforce burnout and turnover [15, 16, 19, 22, 23]. Despite the importance of this, many current health professionals have not had formal training in interprofessional collaboration [24–26]. In moving towards interprofessional team-based practice in the primary care setting, more explicit interprofessional education (IPE) and training can increase positive attitudes and readiness for team-based care [27].

Graduate education for physicians and physician assistants (PAs) in primary care is important for fostering career development and enhancing professional skills to enhance patient care. Unfortunately, clinicians are seldom formally trained in leadership, evaluation, education, or the nuances of a transformed health care system [13, 26, 28, 29]. Few published articles describe curricula or outcomes of IPE programs that train physicians alongside PAs, despite PAs playing an increasingly integral role in the primary care workforce [30].

In 2018, the Health Resources and Service Administration (HRSA) began funding Primary Care Training and Enhancement (PCTE) programs, which provided an opportunity to address a need described by the Society of Teachers in Family Medicine and the Physician Assistant Education Association [31]. In this article, we describe the design, curriculum, and outcomes from a fellowship developed with this support as an example of an IPE initiative for community-based primary care physicians and PAs. We had the ambitious goal of designing a fellowship which uses protected learning time to arm practicing primary care physicians and PAs with the knowledge and confidence that would provide a first step towards increasing the pool of effective, adaptable, and engaged primary care champions who are ready to transform their working environments.

## Methods

### Setting and leadership

The Community Primary Care Champions (CPCC) Fellowship in Cincinnati, Ohio was developed in this context and brought together faculty from the University of Cincinnati College of Medicine Department of Family and Community Medicine and the Mount St. Joseph (MSJ) University Department of Physician Assistant Studies to provide a unique learning opportunity for community-based primary care physicians and PAs. The timing of this initiative was ideal; the MSJ Department of PA Studies was created in 2017 to address a regional health workforce shortage and a lack of regional PA presence in primary care settings.

The leadership team consisted of the director of the MSJ PA training program (principal investigator), the dean of health sciences at MSJ, the program director of a family medicine residency (fellowship program director), a primary care physician (site principal investigator), a dually-boarded psychiatrist and primary care provider (mental health curriculum faculty lead), a PA who is a graduate of the first cohort of fellows (fellowship associate program director), and principal research assistant (project manager). It was important to our team that a fellowship focused on training physicians alongside PAs had interprofessional leadership, brought in speakers with varied professional backgrounds to teach the fellows, and that curricular activities allowed fellows to interact with and learn from each other.

The CPCC fellowship aimed to create a collaborative learning environment that fostered formation of a community of practice among regional primary care clinicians who had the potential to become change agents to promote primary care transformation. The essential elements of a community of practice include 1) a domain (shared interest, goal, or topic; here, primary care transformation), 2) a community (group of individuals that longitudinally and intentionally builds relationships; here, primary care physicians and PAs) and 3) a practice (a shared repertoire of experiences, tools, guidelines; here, a curriculum that builds on clinical experience obtained in regional primary care settings to develop transformational leadership) [32, 33]. Communities of practice are action-oriented groups that convene with a shared mission to learn through shared educational experiences, collaboration, problem-solving, and information- or experience-sharing [32]. The goal of our fellowship was to facilitate an intellectual space for a community of practice to challenge attitudes while expanding knowledge and confidence for primary care leadership [34].

### Curriculum

**Fig. 1:**
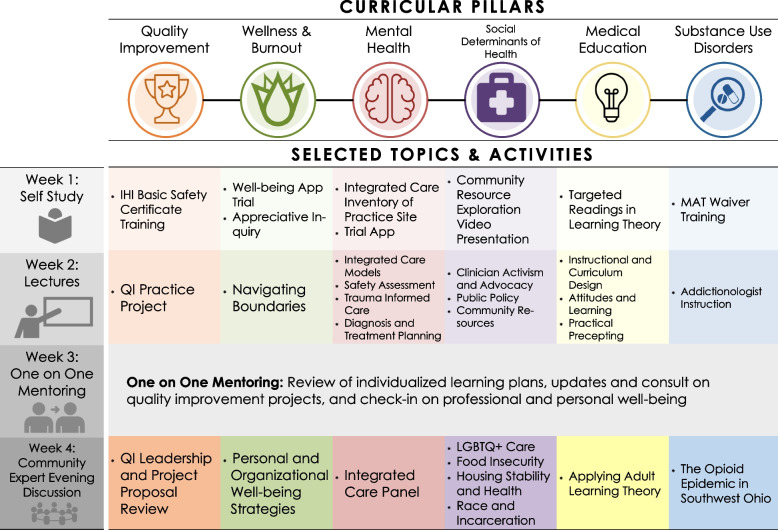
Fellowship curricular pillars with recurring monthly activities

Over the 12-month fellowship, the six pillars of the curriculum addressed regionally relevant themes important for primary care transformation and career development, as seen in Fig. 1. We provided 0.1 FTE (Full Time Equivalency) of protected time to ensure that intellectual space could be dedicated to these foundational topics. Requirements for completion of the fellowship included earning a basic certificate in quality and safety through the Institute for Healthcare Improvement’s Open School, completing an individual QI project related to one of the curricular areas within each fellow’s professional role, and completion of buprenorphine waiver training. At the start of the fellowship, fellows designed an individualized learning plan to optimize their efforts throughout the year and were provided monthly individual coaching from the program director.

Half of the fellowship calendar was devoted to structured self-directed education (assigned readings, modules, reflections, and practical assignments), and half involved participating in group learning activities that engaged the community of practice and enhanced fellows’ cognitive presence (the engagement of critical thinking and intellectual focus on the topic at hand) [35]. To provide a backbone for lessons in IPE, collaboration, and leadership, efforts were made to facilitate discussion among the fellows given their different professional background but similar practice environment, and efforts were made to engage experts from across several professions in learning activities. For instance, a community-based monthly evening conversation provided an opportunity for fellows, fellowship faculty, and regional guest speakers with varied professional backgrounds to discuss a relevant primary care topic within one of the curricular pillars (e.g., food insecurity as a social determinant of health which invited the director of a food bank, a community organizer that runs community gardens, and the director of a school lunch program and the opioid epidemic which invited a police chief, a public health professional specializing in harm reduction services, and a community advocate with lived experience). The participants shared their experiences and challenges relevant to the topic and coalesced practical, actionable ideas to improve the delivery of primary care within their community. Throughout the year, fellows also spent time designing and implementing their QI initiative within their clinical environments, and had an opportunity to problem-solve with regional QI experts during one of the community-based evening sessions.

### Fellow characteristics and recruitment

Eligible fellows were primary care physicians or PAs with at least two years of clinical experience so that the community-based, informal learning they gained in their clinical environment could be applied to the formal learning occurring throughout the fellowship. We recruited via professional networks of the faculty, mentors, and fellows, including statewide and national professional organizations and area-based training programs. The necessary transition to an entirely virtual curriculum in 2020–2022 (due to the pandemic) provided an opportunity to invite fellows from more distant and rural areas to participate, which expanded the geographic/rurality diversity of the fellow cohorts.

As seen in Table 1, twenty-four primary care fellows completed the fellowship over five cohorts (2018–2023; 14 physicians, 9 PAs, 1 behavioralist): three fellows in year 1 (two physicians, one PA), six fellows in year 2 (four physicians and two PAs), five fellows in year 3 (three physicians and two PAs), three fellows in year 4 (two physicians and one PA), and seven fellows in year 5 (three physicians, three PAs, and one behavioralist)Our team used an endowment to welcome a behavioralist working within a primary care residency program in our final cohort.

**Table 1:** Fellow demographics

n *(% of total fellows)*	**Physicians**	**Physician Assistants**	**Total**
**Fellow Title**^a^	14 *(58.3%)*	9 *(37.5%)*	24^a^
**Gender**			
Male	4 *(16.7%)*		4 *(16.7%)*
Female	10 *(41.7%)*	9 *(37.5%)*	20^a^*(83.3%)*
**Race**			
White/European Descent	10 *(41.7%)*	7 *(29.2%)*	18^a^*(75.0%)*
Black/African Descent	2 *(8.3%)*		2 *(8.3%)*
Asian/Asian Descent	3 *(12.5%)*	2 *(8.3%)*	5 *(20.8%)*
**Background**			
Rural	1 *(4.2%)*	4 *(16.7%)*	5 *(20.8%)*
Disadvantaged	1 *(4.2%)*	1 *(4.2%)*	2 *(8.3%)*

^a^One fellow who was a behavioralist is included in these counts

### Evaluation plan

**Fig. 2:**
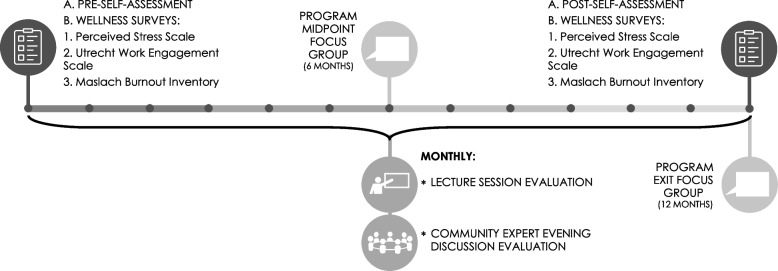
Yearly fellowship evaluation activities

As seen in Fig. 2, evaluation of the fellows included a self-assessment completed at matriculation and the conclusion of the fellowship to assess changes in knowledge, self-efficacy, attitudes, and behaviors related to the objectives of each of the six curricular areas using a five-point Likert scale. One year after graduation from the fellowship, graduates were sent another survey which assessed persistence of knowledge, self-efficacy, attitudes and behaviors related to select fellowship objectives as chosen by the leadership team based on those which seemed most reflective of a transformative experience. Additionally, the wellness of the fellows was assessed at the beginning and conclusion of the fellowship using three validated tools: the Cohen Perceived Stress Scale [36], Ultrecht Work Engagement Scale [37], and the Maslach Burnout Inventory [38]. Fellows were also asked about new leadership or career opportunities arising from completing the fellowship to one year after graduation. Lastly, given the IPE focus of the fellowship, we specifically asked fellows to rate their confidence in working in interprofessional teams in a clinical setting using a five-point Likert scale.

We evaluated the fellowship regularly by having fellows provide feedback on a survey about the content covered and presenters during lectures and evening discussions. In addition, focus groups moderated by the evaluation team halfway through their fellowship and at the end of it provided qualitative feedback on the fellowship structure, content, and support. Results from lecture/evening discussion surveys and the twice-yearly focus groups were analyzed annually and used to continually optimize fellow experience and both effectiveness and meaningfulness of the content and structure of the fellowship.

All survey data was collected via paper copy and in SurveyMonkey. The evaluation plan was approved and determined to be not human subjects research by the University of Cincinnati and Mount St Joseph University Institutional Review Boards.

### Analysis

Fellows’ mean change in self-assessment scores for key knowledge and confidence between matriculation and completion of the fellowship, and between matriculation and one year after completion of the fellowship, were calculated using non-parametric tests with SPSS statistical software. The Rosenthal test was used to measure effect sizes [39]. Statistical significance was set at *p* < 0.05.

The focus groups were transcribed verbatim and analyzed using inductive thematic analysis. Three team members familiarized themselves with the data and one generated initial codes using an iterative process, which were organized into a codebook [40]. Then, three team members used the codebook to code the entire transcript with disparate codes resolved through discussion. The team members then organized codes into unifying themes and subthemes by looking for patterned meaning and interconnections between recurrent features in the codes [41, 42].

## Results

### Self-assessments

**Fig. 3:**
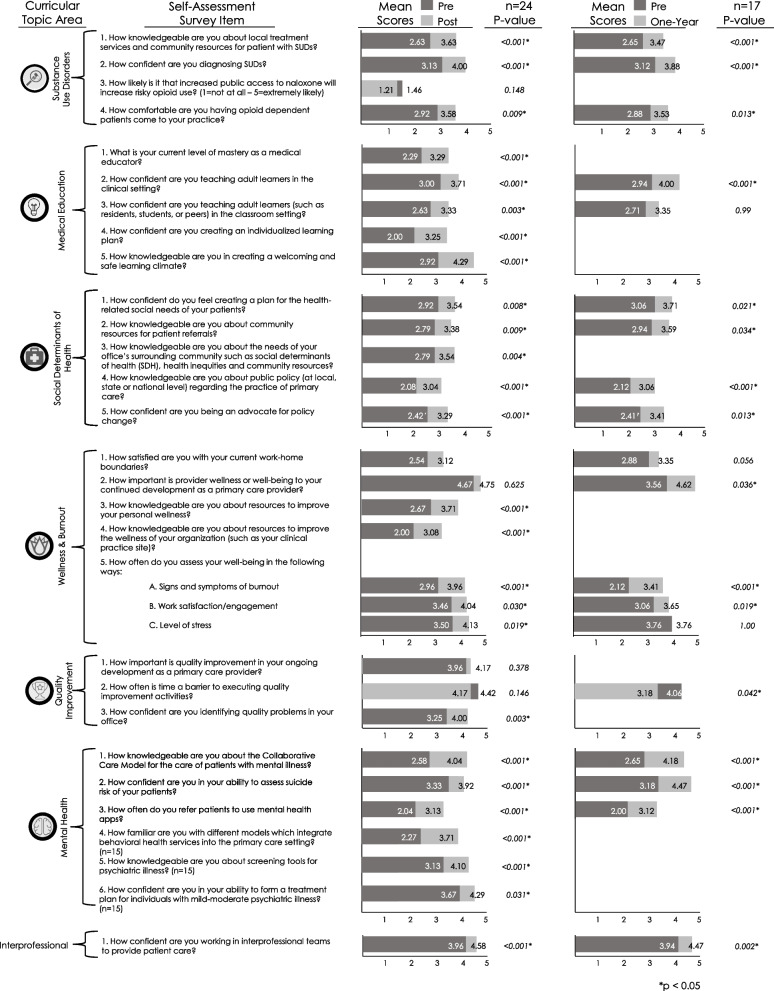
Fellow self-assessment survey results

Fellows’ self-assessments showed a statistically significant increase (*p* < 0.05) in their self-reported knowledge and confidence in most of the fellowship objectives (24 of 28; 85.7%) and all curricular areas at the conclusion of the fellowship, as seen in Fig. 3. Comparing fellows’ self-assessments between matriculation and one year after completion of the fellowship showed statistically significant increase in their knowledge and confidence in 16 of 18 (84.2%) fellowship objectives analyzed, spanning all curricular pillars, indicating persistence of benefit in these areas. In line with our IPE goals, fellows had a significant increase in their own confidence in working in interprofessional teams within the clinical setting, which persisted 12 months after graduation from the fellowship. There was no significant difference in self-assessment scores when comparing physician and PA fellow response, nor in pre- and post-fellowship changes in knowledge and confidence between physicians and PAs.

### Wellness and burnout

**Fig. 4:**
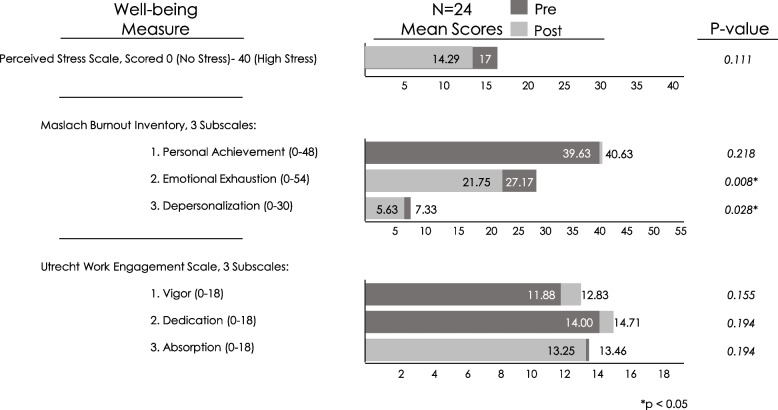
Fellow well-being measure results

Clinician wellness was one of the six curricular pillars; relevant content in didactic lectures and evening discussions included burnout, assessing and optimizing personal and professional wellness, and organizational wellness. Fellow self-assessment surveys demonstrated that self-reported knowledge and confidence in assessing and addressing personal and professional well-being significantly improved. As seen in Fig. 4, fellow surveys assessing stress, work engagement, and burnout showed significant improvement in depersonalization and emotional exhaustion (Maslach Burnout Inventory subscales) but no significant changes in perceived stress, personal achievement, or work engagement between matriculation and conclusion of the fellowship. Twelve (50%) of the fellows reported having new leadership or advocacy roles since matriculation into the fellowship. These roles include practice QI champion leadership, residency program directorship, expanded teaching curriculum and topics, medical education course directorship, and development and leadership of a center for patients with developmental disabilities.

### Fellows’ primary care transformation quality improvement projects

**Fig. 5:**
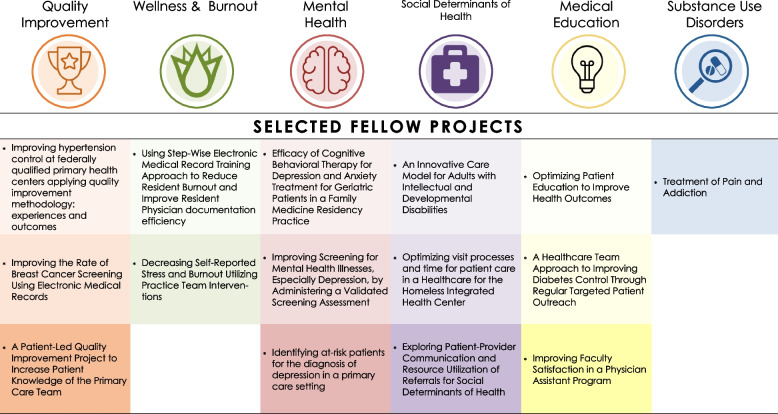
Select primary care transformation quality improvement projects by fellows

Each of the 24 fellows completed a primary care transformation QI project related to one of the fellowship’s curricular pillars. As seen in Fig. 5, topics included educating practice teams on pain and addiction, improving breast cancer screening rates, decreasing resident burnout by teaching documentation efficiency strategies, improving completion rates of depression screenings, and implementing an integrated care model for patients with disabilities. Each fellow was required to draft an abstract of their project for the HRSA annual meeting for an opportunity to present their project virtually or in-person. All fellows were also encouraged to submit project abstracts to other national conferences for presentation; four project abstracts were submitted to non-HRSA conferences, with all four accepted for presentation.

### Qualitative feedback

Feedback for the fellowship was overall positive in fellow focus groups. Specifically, as seen in Table 2, fellows confirmed the relevance of the content included as curricular pillars in their professional development and expressed an appreciation for the variety of topics. Some fellows felt that they started the fellowship with a high level of knowledge and confidence in one pillar (i.e. mental health) but that they had an opportunity to grow in another (i.e. medical education). Overall, each pillar was specifically named as having been an important opportunity for professional growth by fellows in focus groups.

**Table 2:** Qualitative themes and sub-themes from fellow focus-groups eliciting feedback on fellows’ experiences

Themes	Sub-Themes	Exemplar Quotes
Each curricular pillar was relevant to fellows’ careers and contributed to meaningful professional development	The variety of topics allowed for individualized learning	“[The lectures] were just really applicable and easy to understand and engaging. I’ve referenced a lot of the materials provided to us in practice.” “Just having a different outlook on, access to food, access to transportation, homelessness, all those things that we talked about. I just really learned so much more about, because that was one thing that in school that we didn't really touch on at all.” “It helped for me to have some tools to actually figure out how do I best work with learners. It was definitely helpful.” “I was feeling very lackluster in my teaching skills and I've been taking every single tool to heart and using them on my residents, the medical students, fellows. It's been fantastic.”
The format the fellowship allowed for flexibility and collaborative learning among a group of busy professionals		“We gleaned so much information from each other. That's been one of the things I've enjoyed most about this. It's like the true meaning of the term fellowship. We all are primary care to some degree and we have different practices but we have a lot in common.” “Just actually just getting to see each other and share experiences of difficult patient encounters, that was actually really good for my wellness.” “For me it was helpful to have the modules that I could piece here and there and in-between and then to have the flexibility of the self-study time to move things around.”
The inclusion of community members in fellowship activities allowed for expanded perspectives and personal and professional growth for fellows	The highest-rated activity of the fellowship were the evening gatherings which featured topical experts from the community	“It just opened my eyes a little bit more to some of the challenges and the bureaucracy within food banks. I never would have thought about that before.” “I do like the community aspect; different elements of the community and the value of how that can impact primary care, sometimes in non-clinical ways.” “Just hearing about the lived experiences [of those with addiction] was more helpful to me almost than resources. [I] was better able to understand what led them down that path or the difficulties that they face, how I can just better understand them and how maybe what we're doing is helping them or not helping them or their perception on things. And so that was really impactful for me and helped reorient my beliefs or my thoughts on that.” “This fellowship motivated me to want to do more advocacy”

The structure for teaching and learning incorporated a mixture of self-directed asynchronous learning, didactic learning, and collaborative discussions; in focus groups fellows described appreciating the flexibility in teaching and learning methods, given how busy they were. Fellows also expressed an appreciation for the collaborative learning opportunities and requested more opportunities to learn from each other within the community of practice (particularly when the fellowship was virtual due to the pandemic).

Lastly, the fellows described clearly that the evening sessions, which invited interprofessional experts from the community to help teach a topic, contributed to both their personal and professional growth. Specifically, many fellows cited an evening session in which a harm reduction public health expert, a local police chief, and a peer support specialist were invited to discuss strategies for combatting the opioid epidemic were particularly impactful in inspiring changed perspectives on addiction and the role of primary care clinicians.

## Discussion

The CPCC fellows demonstrated improved self-reported knowledge and confidence in topic areas of all six curricular pillars, many of which persisted one year after graduation from the fellowship. Twelve of the 24 fellows also reported having new leadership roles during or after their participation in the fellowship, several of which were relevant to curricular pillars (i.e. practice QI champion leadership, teaching roles). All fellows completed a primary care transformation QI project in their clinical sites relevant to the curricular pillars, and several were able to present these efforts at national conferences. Importantly, our intentional focus on IPE contributed to fellows having improved confidence in working within interprofessional clinical teams, which persisted even a year after graduation from the fellowship.

The fellowship was unique in its inclusion of PAs and physicians within fellow cohorts and the fellowship leadership team. This interprofessional collaboration happened at a critical time in our area, when these two professional groups are increasingly collaborating in the primary care setting due to the opening of the PA program at MSJ, our area’s first PA school. A report of the first four years of the HRSA-funded PCTE program shows that overall, the ratio of physicians to PAs enrolled as fellows in 19 programs was 4:1; in our CPCC fellowship, having PA educators as leadership boosted intentional recruitment of PAs resulting in a more even ratio of 1.6:1 [43].

Critical to the fellowship’s success were its content, structure, and inclusion of interprofessional regional leaders/experts in discussion- and community-based evening learning sessions. There are only three other published accounts of PCTE fellowships, all of which included mentorship on a primary care transformation project as central to the fellowship goals. Lewis et al. (2023) described fellowship content focusing on population health, health care transformation, leadership skill development, interprofessional practice, social determinants of health, and cultural bias and results [44]. Ervin et al. (2023) described a focus on leadership and team communication for their fellowship [45]. Casola et al. [46] described a focus on enhancing practice management and leadership skills while providing training on preceptorship and QI [46]. While most PCTE fellowship programs have not yet published their curriculum or activities, our structure and evening interprofessional community leader discussions appear to be unique among PCTE fellowships. Our CPCC fellowship structure was similar to that of the Kraft Center for Community Health Leadership’s Practitioner program, which trained primary care physicians and nurse practitioners using web-based asynchronous learning, live didactic teaching, discussions, pre-reading of select texts, and mentorship through a quality improvement project,they similarly described participants presenting scholarship to national audiences and an increase in leadership positions amongst graduates [47].

Another impact our fellowship demonstrated was improvement in fellows’ emotional exhaustion and depersonalization, but not in stress, work engagement, or personal achievement. At baseline, the scores for our fellows were similar compared to other published reports on clinician burnout [11, 12]. Given that burnout is driven by systems-level factors rather than individual factors, the high rates of burnout and limited improvement in fellows’ wellness indicators even after training in individual and organizational wellness further proves the importance of training leaders in primary care transformation.

Our evaluation was limited by our use of self-assessment to determine the fellowship’s impact on knowledge and confidence. Also, longitudinal evaluation of some content areas of the fellowship was impacted by our adaptation to the yearly curriculum based on feedback. For instance, new topics such as anti-racism and lesbian, gay, bisexual, transgender, and queer health were added in the last two years on request; limited data is available on the impact of these additions and on fellow outcomes. Finally, while we centered the importance of interprofessional collaboration in the design of our leadership team and curricular activities, we did not directly incorporate evaluation of the impact of IPE on fellows’ knowledge or confidence in interprofessional collaboration until year 4 of the fellowship, which led to limited data being available. An important limitation of the fellowship itself which challenges its sustainability lies in funding; the 0.1 FTE we provided was critical to recruiting fellows and was unavailable at the conclusion of the grant funding period.

Future initiatives should continue to incorporate additional interprofessional members of the primary care team, and community, into professional development opportunities for primary care clinicians. For instance, incorporating pharmacists, nurses, nurse practitioners, and social workers could provide meaningful opportunities for primary care transformation. In addition, having fellows who come from the same clinical site so that they can collaborate on QI initiatives could improve the depth of interprofessional learning that occurs within a similar fellowship. The impact of the interprofessional nature, and preparedness of the learners to work in interprofessional teams, should be directly assessed to better understand how this impacts learning compared to experiences which only invite members of a single profession.

## Conclusions

The CPCC fellowship is an example of an effective IPE learning experience that prepared post-graduate primary care PAs and physicians to be change agents in leading primary care transformation while improving emotional exhaustion and personal achievement. Fellows appreciated the six curricular content areas (QI, wellness and burnout, mental health, the social determinants of health, medical education, and substance use disorders) and demonstrated an improvement in knowledge, attitudes, and confidence in each content area; much of this impact persisted on re-assessment a year after graduation. The structure of the fellowship allowed for the creation of a community of practice that was engaged with the surrounding communities in which they worked. Evening sessions which incorporated interprofessional experts and leaders from the community were particularly impactful and served to challenge pre-existing attitudes and contribute to personal and professional growth.

## Data Availability

The dataset supporting the conclusions is available from the corresponding author on reasonable request.

## Abbreviations

PCMH: Patient centered medical home
CPC Program: Comprehensive Primary Care Program
QI: Quality improvement
IPE: Interprofessional education
PA(s): Physician assistant(s)
HRSA: Health Resources and Service Administration
PCTE: Primary Care Training and Enhancement
CPCC Fellowship: Community Primary Care Champions Fellowship
MSJ: Mount St. Joseph University

## References

[1] Meyers DS (2009). Primary care: too important to fail. Ann Intern Med.

[2] National Association of Community Health Centers. Community Health Center Chartbook. Bethesda: National Association of Community Health Centers; 2023 Mar. Available from: https://www.nachc.org/resource/community-health-center-chartbook-2022/. Cited 2023 Nov 10.

[3] IHS Markit Ltd. The Complexities of Physician Supply and Demand: Projections From 2019 to 2034. Washington DC: Association of American Medical Colleges; 2021. Available from: https://www.aamc.org/data-reports/workforce/data/complexities-physician-supply-and-demand-projections-2019-2034. Cited 2023 Nov 9.

[4] Meredith LS, Bouskill K, Chang J, Larkin J, Motala A, Hempel S (2022). Predictors of burnout among US healthcare providers: a systematic review. BMJ Open.

[5] Hoff T, Trovato K, Kitsakos A. Burnout Among Family Physicians in the United States: A Review of the Literature. Qual Manag Health Care. 2023; Available from: https://journals.lww.com/10.1097/QMH.0000000000000439. Cited 2023 Nov 10. 10.1097/QMH.0000000000000439 37817317

[6] National Center for Health Workforce Analysis. Physician Workforce: Projections, 2021-2036. U.S. Department of Health and Human Services Health Resources and Services Administration; 2023 Oct. Available from: https://bhw.hrsa.gov/sites/default/files/bureau-health-workforce/physicians-projections-factsheet-10-23.pdf. Cited 2023 Nov 10.

[7] Bodenheimer T, Sinsky C (2014). From triple to quadruple aim: care of the patient requires care of the provider. Ann Fam Med.

[8] Center for Medicare & Medicaid Innovation. Findings at a Glance: Synthesis of Evaluation Results across 21 Medicare Models 2012-2020. Baltimore: Center for Medicare & Medicaid Innovation; 2022. Available from: https://www.cms.gov/priorities/innovation/data-and-reports/2022/wp-eval-synthesis-21models-aag. Cited 2023 Nov 10.

[9] Agency for Healthcare Research and Quality. Patient Centered Medical Home (PCMH). Rockville: Agency for Healthcare Research and Quality; 2022 Aug. Available from: https://www.ahrq.gov/ncepcr/research/care-coordination/pcmh/index.html. Cited 2023 Nov 10.

[10] Farrell TW, Greer AG, Bennie S, Hageman H, Pfeifle A (2023). Academic health centers and the quintuple aim of health care. Acad Med.

[11] Soares JP, Lopes RH, Mendonça PBDS, Silva CRDV, Rodrigues CCFM, Castro JLD (2023). Use of the Maslach burnout inventory among public health care professionals: scoping review. JMIR Ment Health.

[12] Atanes ACM, Andreoni S, Hirayama MS, Montero-Marin J, Barros VV, Ronzani TM (2015). Mindfulness, perceived stress, and subjective well-being: a correlational study in primary care health professionals. BMC Complement Altern Med.

[13] McNellis RJ, Genevro JL, Meyers DS (2013). Lessons learned from the study of primary care transformation. Ann Fam Med.

[14] Center for Medicare & Medicaid Innovation. CPC+ 2019 Year in Review [Internet]. Center for Medicare & Medicaid Services; 2019. Available from: https://innovation.cms.gov/innovation-models/comprehensive-primary-care-plus. Cited 2022 Sep 9.

[15] Abraham CM, Zheng K, Poghosyan L (2020). Predictors and outcomes of burnout among primary care providers in the United States: A systematic review. Med Care Res Rev.

[16] Zubatsky M, Pettinelli D, Salas J, Davis D (2018). Associations between integrated care practice and burnout factors of primary care physicians. Fam Med.

[17] White N (2021). Reducing primary care provider burnout with pharmacist-delivered comprehensive medication management. Am J Lifestyle Med.

[18] Liu M, Wang J, Lou J, Zhao R, Deng J, Liu Z (2023). What is the impact of integrated care on the job satisfaction of primary healthcare providers: a systematic review. Hum Resour Health.

[19] Olayiwola JN, Willard-Grace R, Dubé K, Hessler D, Shunk R, Grumbach K (2018). Higher perceived clinic capacity to address patients’ social needs associated with lower burnout in primary care providers. J Health Care Poor Underserved.

[20] Holmes A, Chang YP (2022). Effect of mental health collaborative care models on primary care provider outcomes: an integrative review. Fam Pract.

[21] Pascucci D, Sassano M, Nurchis MC, Cicconi M, Acampora A, Park D (2021). Impact of interprofessional collaboration on chronic disease management: Findings from a systematic review of clinical trial and meta-analysis. Health Policy.

[22] Savageau JA, Ferguson WJ, Bohlke JL, Cragin LJ, O’Connell E (2011). Recruitment and retention of primary care physicians at community health centers: a survey of massachusetts physicians. J Health Care Poor Underserved.

[23] Sinsky CA, Willard-Grace R, Schutzbank AM, Sinsky TA, Margolius D, Bodenheimer T (2013). In search of joy in practice: a report of 23 high-functioning primary care practices. Ann Fam Med.

[24] Price D, Howard M, Hilts L, Dolovich L, McCarthy L, Walsh AE (2009). Interprofessional education in academic family medicine teaching units. Can Fam Physician.

[25] Peccoralo LA, Callahan K, Stark R, DeCherrie LV (2012). Primary care training and the evolving healthcare system: primary care training and evolving the healthcare system. Mt Sinai J Med J Transl Pers Med.

[26] Miller R, Weir C, Gulati S (2018). Transforming primary care: scoping review of research and practice. J Integr Care.

[27] El-Awaisi A, Awaisu A, Aboelbaha S, Abedini Z, Johnson J, Al-Abdulla SA (2021). Perspectives of healthcare professionals toward interprofessional collaboration in primary care settings in a Middle Eastern Country. J Multidiscip Healthc.

[28] American Hospital Association. Physician Leadership Education. Chicago: American Hospital Association; 2014. Available from: https://www.aha.org/system/files/media/file/2020/02/LeadershipEducation.pdf. Cited 2023 Nov 10.

[29] Advisory Committee on Interdisciplinary, Community-Based Linkages. Transforming Interprofessional Health Education and Practice: Moving Learners from the Campus to the Community to Improve Population Health [Internet]. Rockville: U.S. Department of Health and Human Services Health Resources and Services Administration; 2014. Available from: https://www.hrsa.gov/sites/default/files/hrsa/advisory-committees/community-based-linkages/reports/thirteenth-2014.pdf. Cited 2023 Nov 10.

[30] National Center for Health Workforce Analysis. Primary Care Workforce Projections. U.S. Department of Health and Human Services Health Resources and Services Administration; 2023. Available from: https://bhw.hrsa.gov/data-research/projecting-health-workforce-supply-demand/primary-health. Cited 2023 Nov 10.

[31] Brenneman A, Kruse J (2012). Educating primary care teams for the future introducing the joint PAEA/STFM position statement, “Educating Primary Care Teams for the Future: Family Medicine and Physician Assistant Interprofessional Education. J Physician Assist Educ.

[32] Wenger E (1998). Communities of practice: learning, meaning, and identity.

[33] Wenger E. Communities of practice: an introduction. 2011. Available from: https://scholarsbank.uoregon.edu/xmlui/handle/1794/11736?show=full. Cited 2023 Dec 27.

[34] Miller WL, Cohen-Katz J (2010). Creating collaborative learning environments for transforming primary care practices now. Fam Syst Health.

[35] Garrison DR, Cleveland-Innes M (2005). Facilitating cognitive presence in online learning: interaction is not enough. Am J Distance Educ.

[36] Cohen S, Kamarck T, Mermelstein R (1983). A global measure of perceived stress. J Health Soc Behav.

[37] Schaufeli WB, Bakker AB, Salanova M (2006). The measurement of work engagement with a short questionnaire: a cross-national study. Educ Psychol Meas.

[38] Maslach C, Jackson SE (1981). The measurement of experienced burnout. J Organ Behav.

[39] Rosnow RL, Rosenthal R (2003). Effect sizes for experimenting psychologists. Can J Exp Psychol Rev Can Psychol Expérimentale.

[40] Srivastava P, Hopwood N (2009). A practical iterative framework for qualitative data analysis. Int J Qual Methods.

[41] Braun V, Clarke V (2006). Using thematic analysis in psychology. Qual Res Psychol.

[42] Braun V, Clarke V. Thematic Analysis: A Practical Guide. Los Angeles: SAGE Publications Ltd; 2021. p. 376.

[43] U.S. Department of Health and Human Services Health Resources and Services Administration National Center for Health Workforce Analysi. Primary Care Training and Enhancement - Training Primary Care Champions Evaluation. Rockville: U.S. Department of Health and Human Services Health Resources and Services Administration; 2023. Report No.: Academic Years 2018-2022. Available from: https://bhw.hrsa.gov/sites/default/files/bureau-health-workforce/funding/pcte-tpcc-evaluation-report-2018-2022.pdf. Cited 2023 Nov 9.

[44] Lewis JH, Appikatla S, Anderson E, Glaser K, Whisenant EB (2023). The primary care transformation executive fellowship to develop community health center leaders. Adv Med Educ Pract.

[45] Ervin C, Rachel SA, Baker LJ, Joseph L, Roberson D, Omole F (2023). Practical applications of implementing integrated mental health practices with primary care providers. Am Psychol.

[46] Casola AR, Cunningham A, Crittendon D, Kelly S, Sifri R, Arenson C. Implementing and Evaluating a Fellowship for Community-Based Physicians and Physician Assistants: Leadership, Practice Transformation, and Precepting. J Contin Educ Health Prof. 2022;Publish Ahead of Print. Available from: https://journals.lww.com/10.1097/CEH.0000000000000427. Cited 2022 Sep 9. 10.1097/CEH.0000000000000427 35604663

[47] Shtasel D, Hobbs-Knutson K, Tolpin H, Weinstein D, Gottlieb GL (2015). Developing a pipeline for the community-based primary care workforce and its leadership: the kraft center for community health leadership’s fellowship and practitioner programs. Acad Med.

